# New Strategies for Improving Budesonide Skin Retention

**DOI:** 10.3390/pharmaceutics14010030

**Published:** 2021-12-24

**Authors:** Cristina Padula, Ian Pompermayer Machado, Aryane Alves Vigato, Daniele Ribeiro de Araujo

**Affiliations:** 1 Department of Food and Drug, University of Parma, 43100 Parma, Italy; cristina.padula@unipr.it; 2 Department of Fundamental Chemistry, Institute of Chemistry, University of São Paulo, Sao Paulo 01000-000, SP, Brazil; ianpmachado@hotmail.com; 3 Drug and Bioactives Delivery Systems Research Group (SISLIBIO), Human and Natural Sciences Center, Federal University of ABC, Av. dos Estados 5001, Bl A, T 3, Lab 503-3, Bangu, Saint Andrew 090210-580, SP, Brazil; aryanevigato@gmail.com

**Keywords:** budesonide, poloxamers, dermal, skin delivery, atopic dermatitis

## Abstract

The aim of this work was to evaluate the ex vivo effect of the combination of two strategies, complexation with cyclodextrin, and poloxamer hydrogels, for improving water solubility in the dermal absorption of budesonide. Two hydrogels containing 20% poloxamer 407, alone or in combination with poloxamer 403, were prepared. Each formulation was loaded with 0.05% budesonide, using either pure budesonide or its inclusion complex with hydroxypropyl-β-cyclodextrin, and applied in finite dose conditions on porcine skin. The obtained results showed that for all formulations, budesonide accumulated preferentially in the epidermis compared to the dermis. The quantity of budesonide recovered in the receptor compartment was, in all cases, lower than the LOQ of the analytical method, suggesting the absence of possible systemic absorption. The use of a binary poloxamer mixture reduced skin retention, in line with the lower release from the vehicle. When the hydrogels were formulated with the inclusion complex, an increase in budesonide skin retention was observed with both hydrogels. Poloxamer hydrogel proved to be a suitable vehicle for cutaneous administration of budesonide.

## 1. Introduction

Atopic dermatitis (AD), also known as eczema, is one of the most common inflammatory skin disorders and characterized by a broad variety of symptoms, including pruritus, edema, lichenification, and xerosis [1], affecting an average of 12.6% of the worldwide pediatric population (6 month–18 years old) [2]. AD is a complex and multifactorial disease. Although the mechanisms involved in the AD pathophysiology have become better understood in recent years, clinical management is limited to controlling inflammation and improving symptoms. Mild or moderate are the most common forms of AD in the pediatric population [2], but in some patients it may evolve to a more severe form, requiring systemic treatment. In those cases, the first biologic drug, dupilumab, which is a monoclonal human antibody targeting the IL-4 and IL-13 receptor, was made available in 2017 for long-term treatment in adults and adolescents [3,4]. In all other cases, management is based on topical therapies with topical corticosteroids (TCs); topical calcineurin inhibitors, such as picrolimus and pimecrolimus; or with topical crisaborole, a phosphodiesterase 4 inhibitor [5]. Among these options, the mainstay therapy for AD is still considered treatment with TCs, drugs that have been used for more than 60 years [6] and that have proven to be more effective compared to the other therapeutic options for the treatment of moderate to severe AD [5]. Nevertheless, the prolonged use of TCs may be associated with adverse effects, both local (i.e., skin atrophy, teleangiectases, rosacea) and systemic (i.e., pituitary–adrenal axis suppression, hyperglycemia, glaucoma). Systemic adverse effects are uncommon, but they are particularly risky for children, due to their higher skin surface-total body mass ratio compared to adults [7]. This contributes to the diffusion of TC-phobia and to the poor adherence to topical therapy [8]. The current strategy to reduce the risk of side effects of TCs in children is based on the use of TCs with low bioavailability and potency [5,9,10], although a short-duration approach with TCs with a higher potency proved to be similarly effective, with no increase in adverse effects [11]. From this perspective, the use of a potent TC with a low systemic absorption could be a valid alternative.

Budesonide is a potent [10,12,13], non-halogenated acetal corticosteroid. Its low systemic absorption after oral [14] or inhalation [15] administration, as a consequence of an extensive fist-pass effect, makes it a good candidate for treating AD. Presently, budesonide is used in the treatment of asthma [16] and ulcerative colitis [17], but in the early 1980s, its high local anti-inflammatory activity was also successfully exploited for the treatment of skin diseases, such as psoriasis [18,19,20] and atopic dermatitis [21], proving to have a comparable or higher activity with respect to other corticosteroids. More recently, it has been indicated for the clinical management of pediatric atopic dermatitis [10,13]. 

The efficacy and potency of TCs depend on their physicochemical properties and the nature of the vehicle used for topical administration [22,23]. The TCs available on the market are formulated in different vehicles, including ointments, creams, lotions, foams, and gels. Ointment formulations are generally more potent than creams, due to their occlusive properties [24,25], but creams and gels are preferred by the patients because they are cosmetically more appealing. Budesonide is poorly soluble in water [26] and this represents a challenge for the development of formulations. Among the different strategies available, we focused our attention on the use of poloxamers, triblock copolymers composed of hydrophilic (polyethylene oxide), and hydrophobic (polypropylene oxide) blocks that can self-assemble in water when in a concentration higher than the critical micellar concentration and at a certain temperature, named the critical micellar temperature [27]. Due to their amphiphilic nature, the resulting micelles have an hydrophobic core than can solubilize water insoluble molecules [28]. At higher concentrations, some poloxamers form gels in an aqueous medium, which are liquid at low temperature and become gel when the temperature increases, known as the sol–gel transition phenomenon. Poloxamers are a class of copolymers with distinct physicochemical properties, due to their number of polyethylene oxide and polypropylene oxide units, resulting in different polyethylene oxide:polypropylene oxide ratios. This feature confers on them distinct water solubility values and hydrophilic–lipophilic balances (HLB). In this sense, we selected poloxamer 407 and poloxamer 403, with HLB values of 22 and 8, respectively, to form a binary hydrogel matrix with both hydrophilic and hydrophobic components. This type of matrix is particularly useful for balancing the low aqueous solubility of drugs, such as budesonide [29,30]. 

Another interesting strategy is drug complexation with cyclodextrins, in particular the FDA approved hydroxypropyl β-cyclodextrin (HP-β-CD). Complexation with cyclodextrins is a well-known alternative for overcoming physicochemical limitations, such as low aqueous solubility. Thus, a combination of both strategies, drug–cyclodextrin inclusion complex in thermosensitive hydrogels, has been successfully used to formulate budesonide hybrid hydrogels for the treatment of ulcerative colitis [30]. However, to the best of our knowledge, this type of system has not been explored as a skin drug delivery strategy. In particular, we propose this system for achieving both features: enhanced drug aqueous solubility, and skin delivery performance. Since poloxamer gels are easy to prepare and to apply, the drug release to promote or retard skin permeation or accumulation can be modulated [31]. We therefore designed this work to evaluate them as potential matrices for the dermal delivery of budesonide. Particular objectives were the evaluation of the effect of poloxamer composition, hydrogel structural organization, and the use of a budesonide inclusion complex in HP-β-CD on accumulation in the epidermis and dermis.

## 2. Materials and Methods

### 2.1. Reagents

Poloxamer 407 (PL407), Poloxamer 403 (PL403), 2-Hydroxypropyl-β-cyclodextrin (HP-β-CD), and budesonide (BUD) were purchased from Sigma Chem. Comp. (St. Louis, MO, USA). Cetomacrogol 100 was from A.C.E.F. (Fiorenzuola d’Arda, Italy). All other chemicals used were of analytical grade.

### 2.2. Hydrogels Preparation 

First, BUD–HP-β-CD inclusion complexes were obtained by mixing HP-β-CD and BUD in water, at a 1:1 drug:CD molar ratio, for 12 h, at 25 °C, freeze-dried and stored at −20 °C until use [32]. For hydrogel preparation, appropriate amounts of PL407 (18 or 20% *w*/*w*), isolated or in association with PL403 (2 % *w*/*w*), were solubilized in ultrapure water under 4 °C until reaching transparency [33]. BUD or its inclusion complex with HP-β-CD were incorporated into PL-hydrogels at a final drug concentration of 0.05% (*w*/*w*). Table 1 reports the percentage composition of the hydrogels prepared.

**Scheme 1:**
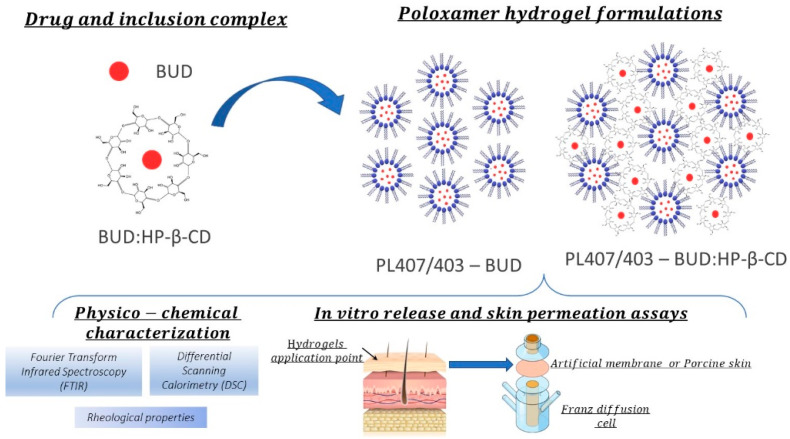
Experimental design for hydrogel physicochemical and in vitro/ex vivo assays.

All formulations were characterized using physicochemical techniques and then evaluated by in vitro release mechanisms and skin permeation profiles, as displayed in Scheme 1:

### 2.3. Fourier Transform Infrared Spectroscopy (FTIR) Assays

Before analysis, the hydrogel samples were dried in a desiccator for at least 12 h. Fourier transform infrared spectra were acquired for hydrogel isolated components (PL407, PL403, BUD, HP-β-CD), BUD/HP-β-CD physical mixture, BUD–HP-β-CD inclusion complex, and dried hydrogel formulations. Samples were placed in a sample holder and analyzed in ATR mode (PerkinElmer Spectrum Two 160000A). Spectra were recorded from 4000 to 650 cm^−1^ with a resolution of 1 cm^−1^.

### 2.4. Differential Scanning Calorimetry: Thermoresponsive Properties Evaluation

Calorimetric assays were carried out by evaluating three successive cycles as heat-cool-heat mode from 0 to 50 °C, at a 5 °C/min heating–cooling ratio (DSC 214—Polyma, Netzsch Instruments). Hydrogels were weighed (25 mg) in aluminum pans and analyzed as described before. An empty aluminum pan was used as a sample reference. Thermograms were represented as flux (k J mol^−1^) against temperature (°C) and all parameters related to micelles formation were determined considering the initial (Tonset), peak (Tpeak), and final (Tendset) temperatures, as well as the enthalpy variation (ΔH°) relative to Tpeak. 

### 2.5. Rheological Studies: Temperature and Frequency Variation

Rheological oscillatory analyses were performed using a rheometer (Kinexus Lab, Malvern Instruments Ltd., England, UK) with a cone-plate geometry (40 mm, diameter). Sol–gel transitions temperatures (Tsol-gel) were determined for each hydrogel before and after BUD, HP-β-CD, or their inclusion-complex incorporation. Hydrogels samples (1 mL) were analyzed under the temperature interval from 10 to 50 °C, for determining mechanical parameters such as the elastic modulus (G′), viscous modulus (G″), and viscosity (η). Additionally, frequency sweep analyzes (0.1 to 10 Hz) were also performed at 32.5 °C and a shear stress of 2 Pa. For data treatment, rSpace software for Kinexus^®^ was used. 

### 2.6. In Vitro Release and Skin Permeation Experiments 

In vitro release and skin permeation experiments were performed using a vertical diffusion cell (Franz type) apparatus, with a diffusional area of 0.6 cm^2^. The cells consisted of a donor compartment, with a volume of approximately 1 mL and a receptor compartment, with a volume of approximately 4 mL separated by a membrane. In the case of the release experiment, an artificial membrane of cellulose acetate with a pore size of 0.45 µm was used. In the permeation experiments, the skin from the outer part of the porcine ears was placed between the receptor and donor compartment, with the stratum corneum facing the donor compartment. In both experiments, the receptor compartment was filled with NaCl 0.9% containing 0.5% *w*/*v* Cetomacrogol 1000, to increase the budesonide water solubility (215 ± 1 μg/)mL; the duration was 6 h and the temperature on the membrane and skin surface was maintained at 32 °C by immersion of the cells in a water bath. 

For the release experiments, the donor was filled with 300 mg/cm^2^ of the formulation to be tested (infinite dose condition). 

For the permeation experiments, the formulation to be tested was applied in finite dose conditions (10 mg/cm^2^) on the skin surface. At the end of the experiment, the cell was dismantled, the skin surface was cleaned twice with cotton pads wet with water, in order to remove the unabsorbed drug, and cut corresponding to the permeation area. The epidermis was heat separated from the dermis, and the skin layers were inserted in pre-weighed test tubes. The amount of tissue was determined by weight. Budesonide accumulated was extracted with 1 mL of acetonitrile:water (70:30, *v*/*v*) at room temperature overnight. In this condition, the extraction was quantitative [34]. The quantification of budesonide in samples was performed using HPLC (Flexar, Perkin Elmer, Waltham, MA, USA) with a reverse-phase Waters Symmetry C18 column (75 × 4.6 mm, 3.5 µm, Waters, Milford, MA, USA). The mobile phase was composed of methanol and water (69:31, *v*/*v*) and was pumped at 0.8 mL/min. UV absorbance was monitored at 254 nm [34].

## 3. Results

### 3.1. Hydrogels Physico-Chemical Characterization

#### Fourier Transform Infrared Spectroscopy (FTIR) Assays

**Figure 1:**
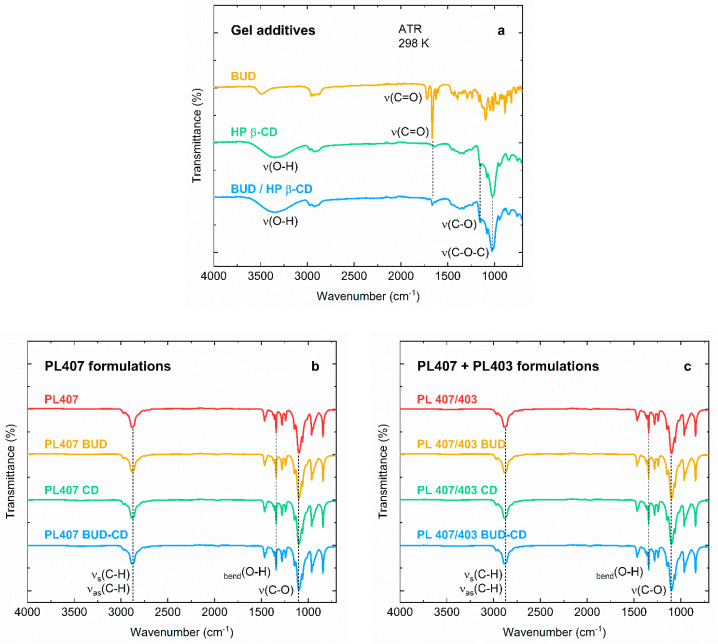
FTIR spectra of the (**a**) gel additives budesonide (BUD), hydroxypropyl β-ciclodextrin (HP-β-CD), and the BUD–HP-β-CD inclusion complex; (**b**) PL407-based formulations; (**c**) PL407/403-based formulations.

The FTIR spectra of hydrogel components are displayed in Figure 1a. The BUD–FTIR spectrum showed the two characteristics bands of carbonyl (C=O) vibration, at 1723 cm^−1^ for the non-conjugated acetyl group, and at 1666 cm^−1^ for the conjugated dihydrobenzenoquinone, as also described by [35]. For HP-β-CD, the FTIR spectrum revealed the main absorption bands attributed to the stretching vibrations of O–H groups (3650–3040 cm^−1^), C–O stretching vibrations (1153 cm^−1^), and C–O–C stretching vibrations (1027 cm^−1^). Additionally, it is possible to observe that the FTIR spectra of the inclusion complex BUD–HP-β-CD and HP-β-CD alone are quite similar, although the two carbonyl bands of BUD can be spotted in the inclusion complex spectrum, with the same wavenumber observed for the BUD alone. This result is indicative that the BUD molecules were encapsulated by HP β-CD, even though no covalent interactions between the two species were formed.

Regarding the hydrogel spectra, they were almost identical, even when comparing formulations containing only PL407 (Figure 1b) and its association with PL403 (Figure 1c). The main bands assigned to the PL molecular structure can be observed at ~2900 cm^−1^ for symmetric and asymmetric C–H stretching vibrations, 1342 cm^−1^ for O–H bending, and at 1100 cm^−1^ for C–O vibrations [PL 1 and 2]. Furthermore, the formation of new peaks was not observed, suggesting a complete dispersion and compatibility of all hydrogels components. Those observations are in agreement with previous reports in the literature for BUD–HP-β-CD inclusion complexes [32] and other corticoids such as triamcinolone [36].

### 3.2. Thermoreversible and Mechanical Properties: Micellization and Sol–Gel Transition Temperatures Are Influenced by Inclusion Complex Incorporation 

A calorimetric analysis was performed to study the micellization process, while the sol–gel transition was studied by rheological analysis, making it possible to determine temperatures relative to the micelles formation (Tonset, Tpeak) and sol–gel transition phenomenon. Additionally, rheological parameters such as elastic (G′) and viscous (G″) moduli, as well as apparent viscosity (η*), were evaluated, to study the influence of all components on the hydrogel mechanical properties. All results are presented in Table 2.

In general, all formulations showed similar micellization temperatures (Tonset and Tpeak), indicating that the incorporation of BUD, HP-β-CD, or their inclusion complex did not shift temperatures relative to the micellization process. However, comparisons among systems composed of PL407 and its association with PL403 revealed reduced Tonset and Tpeak values, which was expected, due to the PL407–403 binary hydrogel formation, since PL403 is a more hydrophobic copolymer (hydrophilic–lipophilic balance-HLB = 8) compared to PL407 (HLB = 22) [37]. Another important point is that the incorporation of BUD or its inclusion complex was able to reduce these temperatures, which can be attributed to the probable BUD insertion into the micellar hydrophobic core and the inclusion complex interaction with hydrophilic corona or hydrogel intermicellar spaces. In fact, the enthalpy variation values were enhanced by BUD and BUD–HP-β-CD, especially for PL407–PL403, indicating that the addition of these components was able to disturb the micelles self-assembly, confirming the BUD and BUD–HP-β-CD interactions with PPG and PEG blocks from the PL structure. 

In a complementary way, rheological analysis allowed Tsol–gel transition determination. This process is a consequence of micelles self-assembly for hydrogel formation in highly concentrated PL systems. In general, the Tsol–gel was quite similar for both hydrogels compositions, even after BUD and its inclusion complex incorporation. All formulations showed a viscoelastic behavior, since the G′ values were higher than that observed for G″. These parameters were compared in terms of the G′/G″ relationships, obtaining values from 5.4 to 8.4 and 5.8 to 7.7, for PL407 and PL407–403, respectively. In particular, the addition of BUD or inclusion complex increased the G′/G″ values, as an indication of the hydrogel structural disturbance caused by these components. 

PL-based hydrogel formation is a process dependent on temperature and polymer concentration, and it is possible to produce systems with high G′/G″ relationships, especially for topical administration purposes, with the advantage of formulation viscosity being modulated by both those conditions. The sol–gel transition process begins when PL micelles are self-assembled and both, temperature and polymer final concentration induce the formation of structurally organized systems, due to PPG-core dehydration and water molecule movement from the hydrophobic core in the direction of the hydrophilic corona [37]. For this reason, the incorporation of additives (salts, drugs, other polymers, etc.) that could potentially interact with the hydrophobic core, or even the hydrophilic corona, can disturb this process. In the case of HP-β-CD incorporation, the interaction with PL can be achieved by two different mechanisms: the complexation of PL hydrophobic units (PPG) with the HP-β-CD apolar cavity, and/or HP-β-CD hydroxyl group interaction with the hydrophilic micellar corona composed of PEO units. Even considering these possible factors, the micellization temperatures were not significantly affected; in agreement with our previous report [32]. On the other hand, shifts were detected from a small displacement in Tsol–gel values, which can be explained by the micelle–micelle interaction disturbance, due to the hydrogen bonds formed by the PEO and hydroxyl groups from PL and HP-β-CD structures, respectively [38]. 

Another important point to consider, is the use of the PL407–403 binary system as an effective combination for drug delivery purposes. Previous studies reported structural analyses, comparing PL with different hydrophilic–lipophilic balances (HLB), such as PL407, PL338, PL188, and PL403. The main findings revealed that drug incorporation induced a cooperative interaction between the hydrophobic and hydrophilic copolymers, for forming mixed micelles able to enhance the incorporation of drugs with low aqueous solubility [32,36,39,40]. In this sense, the insertion of a highly hydrophobic drug, such as BUD (~0.04 mg/mL^−1^, [35]) and its BUD–HP-β-CD inclusion complex shifted either the Tsol–gel or the G′/G″ relationship, but maintained high η* values; even considering the different hydrogel compositions at the same polymer final concentration, which is an important condition, to avoid the application of high flow formulations on the skin. 

### 3.3. In Vitro Release and Skin Permeation Experiments 

**Figure 2:**
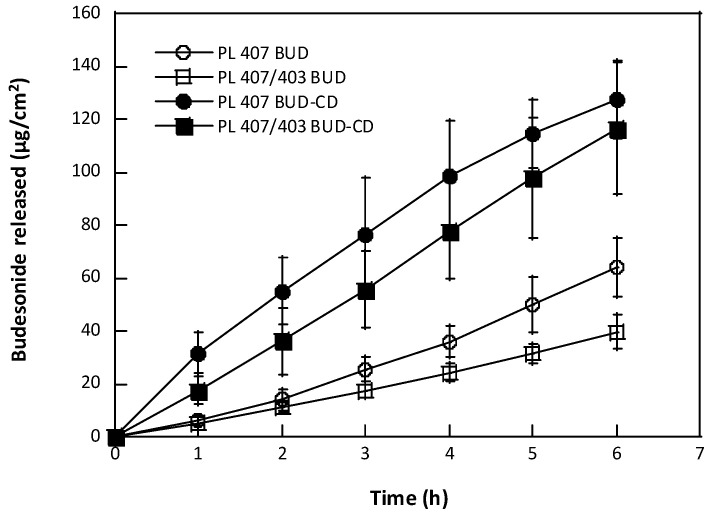
Budesonide release profiles from hydrogels across synthetic membranes (mean value ± sd, n = 6/formulation).

The in vitro release profiles of BUD from the hydrogels formulated, across a synthetic membrane are reported in Figure 2 and Table 3. 

In general, the hydrogels induced a gradual drug release against time, but lower BUD amounts were released from hydrogels containing non-complexed BUD (63.8 and 39.7 µg cm^−2^, for PL407PL403 BUD and PL407 BUD, respectively), when compared to BUD–CD-hydrogels (116.6 and 127.5 µg cm^−2^, for both PL407PL403 CD and PL407 CD), with *p* <0.001 (Figure 2). From the release data, the release constants were then calculated by different mathematical models (Table 3), such as the zero-order, Higuchi, Hixson–Crowel, and Korsmeyer–Peppas. As also observed for the drug release profiles, high release constant (K) values were obtained for hydrogels containing BUD–CD, with best fits determined by the Higuchi model (0.967 ≤ R^2^ ≤ 0.999), indicating that the drug release rate is controlled by a diffusion mechanism from the hydrogel matrix to the medium. Similar results were also reported by [32] when incorporating BUD–CD into PL-based hydrogels for colonic administration. In a complementary way, other studies reported BUD complexation with HP-β-CD and PL407PL403 mixed micelles, for intranasal and pulmonary applications, respectively. Even considering the differences among experimental conditions, the results from those studies reported that the BUD release was controlled by both HP-β-CD and/or PL-based systems [39,40]. 

Those findings indicated that the BUD–CD complex was dispersed into the hydrogel matrix, enhancing the drug aqueous solubility and its capability for achieving release of the medium. In fact, a previous study reported that the BUD aqueous solubility was shifted from 3.9 to 14.6 µg/mL (or 0.009 to 0.034 mM), after complexation with HP-β-CD (1:1 drug:CD molar ratio) [30], contributing to the release profiles described here. Another important point is that, compared to water, both systems, PL407 and the PL407–403 binary mixture, were able to increase the BUD solubility according to the polymer concentration. The binary system proved to be more efficient, with respect to PL407 alone, at the same concentration, indicating that the presence of PL403 played an important role increasing the BUD solubility, due to its lower HLB (8), in relation to PL407 (22) [41], which improved the micellar core hydrophobicity, favoring the incorporation of budesonide [42]. 

**Figure 3:**
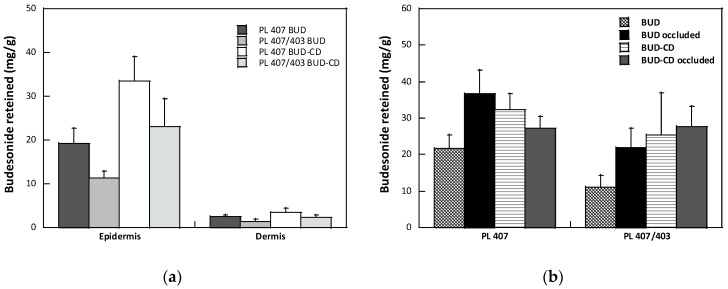
(**a**) Effect of formulation on BUD retention in the epidermis and dermis (mean value ± sem); (**b**) Effect of occlusion on the total amount of BUD recovered from the skin (mean value ± sem).

Ex vivo permeation assays were also performed to compare the formulation performances. All results are summarized in Figure 3.

For all formulations tested, the amount of BUD recovered in the receptor compartment was under the lower limit of quantification of the analytical method (0.05 µg/mL [34]), suggesting that the potential systemic absorption was avoided. As reported in Figure 3a, BUD accumulated preferentially in the epidermis compared to the dermis. The higher accumulation in the epidermis was probably due to the so-called reservoir effect [43], consisting in the formation of a depot of the corticosteroid in the stratum corneum, confirmed by the lower BUD skin retention observed after the partial removal of the stratum corneum by tape stripping (data not shown). The formation of a drug depot is due to the high solubility of corticosteroids in the stratum corneum [44], and this is well described in the literature [22,45,46]. Magnusson and collaborators [47] indicated a log *p* value of 1.4 as the limit for an adequate partition in the stratum corneum of steroids; for values higher than this limit, distribution is favored. The data obtained with budesonide, with a log *p* of 2.32 [48], are in agreement with this hypothesis.

Focusing on the composition of the hydrogels, the presence of a small amount of PL403 (PL407 BUD vs. PL407PL403 BUD) reduced the drug’s skin accumulation into the epidermis. From a general point of view, percutaneous penetration of glucocorticoid can be considered as a sequence of partitioning and diffusion events: the drug is first released from the vehicle, then penetrates and diffuses into the stratum corneum, and after partitioning in the viable epidermis and dermis, diffuses to reach the glucocorticoid receptor. The limiting step in this event sequence is the penetration in the stratum corneum, which is influenced by the physicochemical characteristics of both drug and vehicle. Data from the literature suggest that the enhancement of drug solubility into the vehicle evokes a high drug partition in the stratum corneum and, consequently, the drug bioavailability [45]. As also discussed previously, the presence of a small amount of PL403 increased the BUD solubility, as a result of the formation of a more hydrophobic microenvironment into the micelles core, thus reducing the partitioning and the diffusion of BUD in the vehicle and, consequently, in the stratum corneum.

When the hydrogels were formulated with the inclusion complex in HP-β-CD, an increase in BUD skin retention was observed for both formulations (PL407 BUD vs. PL407 BUD CD, PL407/403 BUD vs. PL407/403 BUD CD). A similar trend was also observed in the case of the release data (Figure 2). The formation of an inclusion complex between a drug and a cyclodextrin is based on an unspecific interaction between the guest and the cyclodextrin’s cavity. Due to the unspecific nature of such interactions, other molecules present in the bulk can compete with the guest to host the CD cavity, with a consequence for the solubility of the guest in the medium [46]. It is reported that surfactants with appropriate characteristics can compete with the guest to host the CD cavity. This displacement can reduce the solubility of the drug in the medium, but if the surfactant molecules are present in an amount higher than their critical micellar concentration, the free drug can be included in the micelle’s core and an increase in solubility can be observed [49], which contributes to explaining the results described here. A similar competitive displacement was observed in the case of triamcinolone acetonide included in methyl-β-cyclodextrin and ciclopirox olamine included in HP-β-CD, by PL407 [50,51]. However, this effect seemed not be determinant for the systems studied here, especially considering the high association constant between BUD and HP-β-CD (Ka = 10,587.8 M^−1^) [30].

Considering the clinical perspective, the management of dermatosis often involves the use of topical corticosteroids in occlusive conditions [52]. Occlusion can be considered the simplest permeation enhancer method and consists in covering the application site with an impermeable dressing. This causes a reduction in transepidermal water loss (TEWL) and an increase in the stratum corneum hydration, which can improve the skin absorption of drugs, although the effect depends on the characteristics of both the drug molecule and the vehicle [53]. The effect of occlusion on the skin accumulation of BUD from the hydrogels proposed is reported in Figure 3b. Data are reported as the total quantity of drug recovered in the epidermis and dermis. In general, the occlusion increased the amount of BUD recovered in the skin for all the formulations tested, except for the PL 407 gel formulated with BUD–HP-B-CD inclusion complex. The high variability of the data, however, does not allow observing significant differences; thus, confirming that occlusion increases the percutaneous absorption of many, but not all, drugs.

## 4. Conclusions

**Figure 4:**
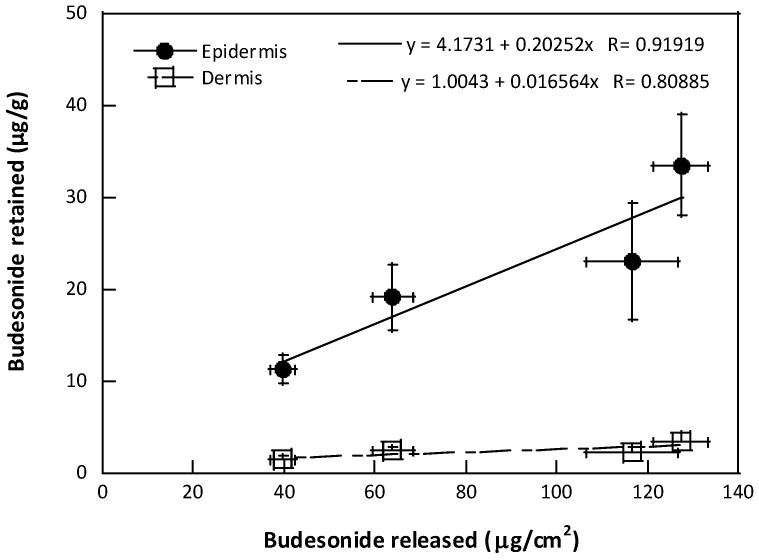
Correlation between the amount of BUD retained in the skin layers and the amount released after 6 h (mean value ± sem).

Hydrogels containing budesonide were formulated using two poloxamers, with distinct HLB ratio values, and with the drug as it is, or as an inclusion complex in hydroxypropyl-β-cyclodextrin. These factors proved to have a role in the delivery profile and accumulation of budesonide in the skin layers, as evidenced by the linear relationship between the amount of budesonide released from the formulation after 6 h and the amount accumulated in the epidermis and dermis after the same time of contact (Figure 4). 

The best performing formulation proved to be the hydrogel containing only PL407 and the budesonide inclusion complex. However, the proposed strategies for improving the dermal delivery of budesonide were shown to be capable of modulating both, the release, and the skin accumulation, avoiding potential systemic drug absorption and, consequently, its side effects.

## Figures and Tables

**Table 1:** Hydrogel percentage composition (*w*/*w*).

Component	PL407 BUD	PL407/403 BUD	PL 407 BUD/HP-β-CD	PL 407/403 BUD/HP-β-CD
Poloxamer 407	20	18	20	18
Poloxamer 403	-	2	-	2
BUD	0.05	0.05	-	-
BUD- HP-β-CD	-	-	0.11 *	0.11 ^1^
Water	Up to 100 g	Up to 100 g	Up to 100 g	Up to 100 g

^1^ Corresponding to 0.05 BUD.

**Table 2:** Values referring to micellization temperatures (Tm), enthalpy (ΔH), G′ (Elastic), and G″ (Viscous) moduli, viscosity (η. mPa s) at 25 °C and 37 °C and sol–gel transition temperatures (Tsol-gel) relative to hydrogels.

Formulations	Tpeak (°C)	ΔH° (J/g)	Tsol–gel (°C)	G′ (10^5^ mPa)	G″ (10^5^ mPa)	η* (10^5^ mPa s) at Tsol–gel
PL407	18.5	3.5	22.7	1235	271.5	2013
PL407-HP-β-CD	17.5	3.7	23.7	1974	403.6	3206
PL407-BUD	18.1	3.5	23.4	2710	381.7	4356
PL407-BUD-HP-β-CD	18.2	3.9	23.3	2156	255.9	3455
PL407-403	16.0	4.2	23.1	3332	571.0	5381
PL407-403-HP-β-CD	16.0	4.5	23.3	3371	431.7	3294
PL407-403-BUD	16.2	5.8	23.1	2853	397.2	4585
PL407-403-BUD-HP-β-CD	16.1	5.1	23.2	3097	403.4	4971

**Table 3:** Budesonide release constants from PL-based hydrogels, according to mathematic models.

Formulation	Zero Order		Higuchi		Hixson–Crowell		Korsmeyer–Peppas		
	K (µg cm^−2^ h^−1^)	R^2^	K (µg cm^−2^ h^−1^)	R^2^	K (µg cm^−2^ h^−1^)	R^2^	K (µg cm^−2^ h^−1^)	R^2^	n
PL407 BUD	11.6 ± 0.6	0.950	39.8 ± 4.5	0.990	69.7 ± 9.4	0.932	1.26 ± 0.04	0.995	0.45
PL407/403 BUD	6.9 ± 0.2	0.947	23.8 ± 2.2	0.967	42.7 ± 4.7	0.952	1.28 ± 0.12	0.965	0.68
PL 407 BUD/HP-β-CD	19.4 ± 1.0	0.989	68.1 ± 2.0	0.996	120.3 ± 5.6	0.991	0.76 ± 0.04	0.989	0.75
PL 407/403 BUD/HP-β-CD	20.1 ± 0.2	0.999	69.6 ± 4.8	0.999	122.4 ± 10.9	0.969	1.03 ± 0.05	0.992	0.22

## Data Availability

Data available on motivated request.
